# Delayed cerebral radiation necrosis after osteoradionecrosis of the skull base: a case report

**DOI:** 10.3389/fonc.2026.1814632

**Published:** 2026-05-21

**Authors:** Maria A. Jacome, Nadia Khalil, Neena Viswanathan, Sheethal Cyriac, Sepideh Mokhtari, Edwin Peguero, Yolanda Piña

**Affiliations:** 1 Department of Neurology, Florida International University Herbert Wertheim College of Medicine, Miami, FL, United States; 2 Department of Neurology, University of South Florida Morsani College of Medicine, Tampa, FL, United States; 3 Department of Neuro-Oncology, H. Lee Moffit Cancer Center and Research Institute, Tampa, FL, United States

**Keywords:** cerebral radiation necrosis, delayed radiation injury, headache, nasopharyngeal carcinoma, skull base osteoradionecrosis

## Abstract

Delayed cerebral radiation necrosis (CRN) is a condition characterized by fibrinoid necrosis, blood-brain barrier disruption, and demyelination due to endothelial cell and oligodendrocyte injury as a result of radiation therapy. The presentation is variable as neurologic manifestations depend on the site of the lesion. This case report describes a rare presentation of delayed CRN years after diagnosis and treatment of osteoradionecrosis of the skull base. It highlights the importance of keeping this diagnosis on the differential for patients with a history of radiation therapy to the head or neck who present with new neurological symptoms and/or radiographic findings, even if prior sites of necrosis were treated and there was no subsequent re-irradiation. This report also underscores how CRN is often difficult to distinguish from tumor progression and how assessment of treatment response may help inform the diagnosis.

## Introduction

Delayed cerebral radiation necrosis (CRN) occurs as a result of endothelial cell and oligodendrocyte injury, perpetuated by a subsequent pro-inflammatory cascade ultimately causing fibrinoid necrosis, blood-brain barrier disruption, and demyelination resulting anywhere from weeks to years after radiation therapy (1, 2). CRN occurs more often after radiation therapy to the head for brain tumors, but it can happen after radiation therapy to the nasopharynx and neck for nasopharyngeal carcinoma, as in the case we present here. The incidence of CRN is dependent on total radiation dose, radiation dose per fraction, targeted volume, type of radiation therapy, concurrent use of chemotherapy, and re-irradiation (2–5). However, with the spread of intensity-modulated radiation therapy (IMRT) there has been a decline of CRN and its extent, attributed to normal tissue sparing techniques (5, 6). Diagnosing radiation necrosis poses a challenge as it could be mistaken as tumor progression. In here, we present a case of a 54-year-old man with a history of nasopharyngeal carcinoma treated with radiation therapy, who presented with fatigue, expressive aphasia, and worsening headaches to the neurology service where he was found to have delayed CRN, 5 years after completing treatment for osteoradionecrosis of the clivus.

## Case report

A 54-year-old right-handed man presented to the Neuro-Oncology department at Moffitt Cancer Center with fatigue, expressive aphasia, and worsening headache.

Seven years earlier, the patient was diagnosed with Epstein-Barr virus-positive nasopharyngeal carcinoma. Patient was a never-smoker with only relevant past medical history of gastroesophageal reflux disease. At the time, he was treated at another facility with 70 Gy external beam radiation therapy (EBRT) to the nasopharynx and neck with concurrent cisplatin for three weeks followed by adjuvant cisplatin/5-fluorouracil chemotherapy for three cycles. Three years after his initial diagnosis he was diagnosed with recurrent focal disease by radiographic surveillance imaging. He was re-irradiated with 40 Gy stereotactic beam radiation therapy (SBRT). Unfortunately, access to these radiation records were never disclosed to us.

**Figure 1:**
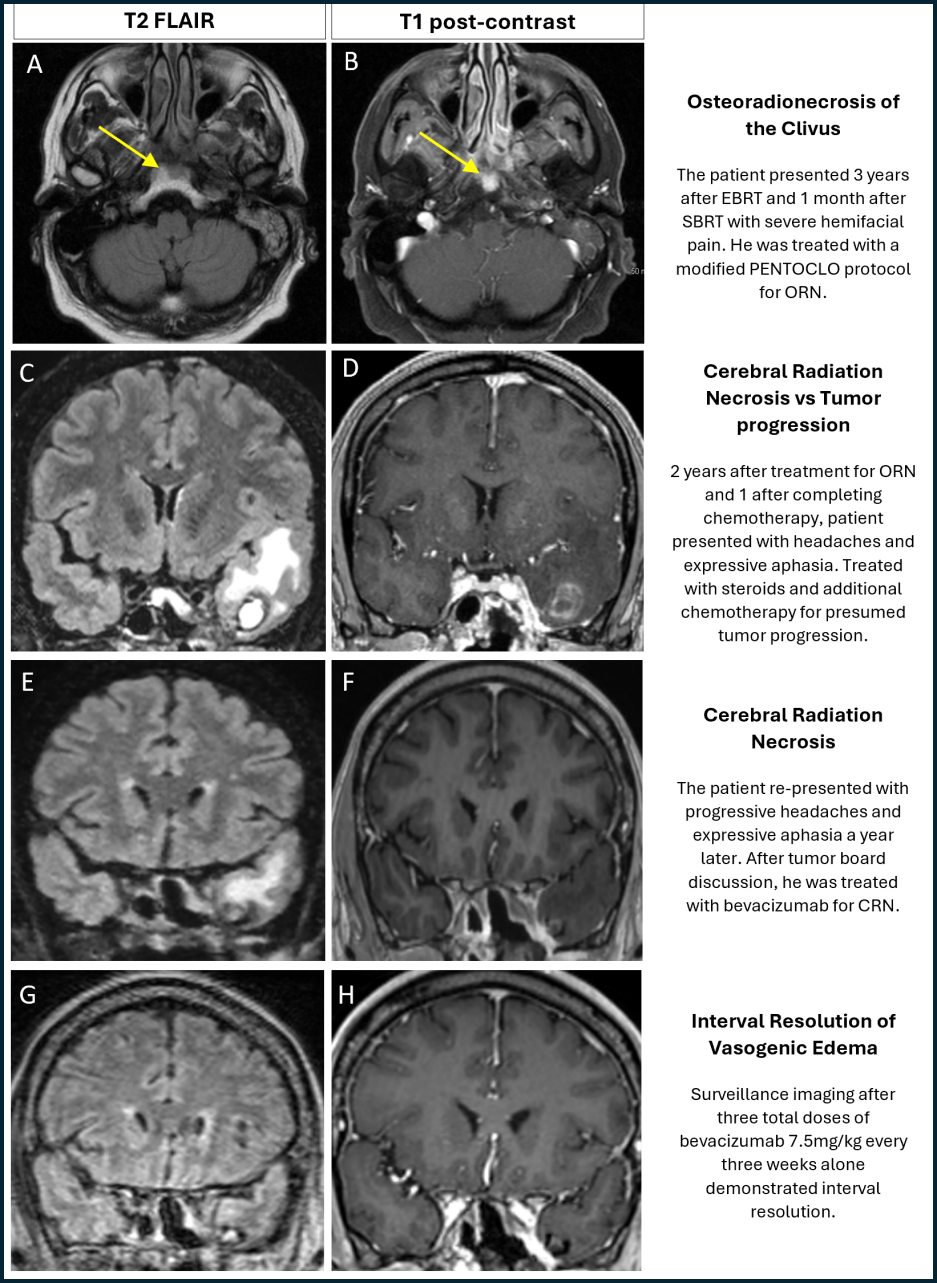
Neuro-imaging depicting osteoradionecrosis, delayed cerebral radiation necrosis, and treatment response. Our patient developed skull base osteoradionecrosis and late radiation necrosis of the temporal lobe, 4 years after treatment as seen in sequential MRI. Initial osteoradionecrosis of the clivus and posterior nasopharynx seen as hypointensity in axial T2 FLAIR sequence (**(A)**, yellow arrow) and enhancement in axial T1 post-contrast [**(B)**, yellow arrow]; patient treated with PENTOCLO protocol. 2 years after treatment for osteoradionecrosis, surveillance MRI showed a 1.6 x 1.3 cm hemorrhagic mass of the left temporal lobe tip with vasogenic edema in coronal T2 FLAIR sequence **(C)** and enhancement in coronal T1 post contrast **(D)**, as well as changes concerning for nasopharyngeal carcinoma progression on the left aspect of nasopharynx and left posterior nasal cavity with tumor involving left pterygopalatine fossa, left skull base, posterior orbit, and left cavernous sinus; treated as tumor progression. Almost two years later patient continued to have symptoms of headache and expressive aphasia, and 0.6 cm and 0.5 cm similar-appearing lesions in the parenchyma of the left temporal lobe with associated vasogenic edema were seen in coronal T2 FLAIR **(E)** and coronal T1 post contrast **(F)** MRI sequences. Nasopharyngeal carcinoma demonstrated stable interval changes, increasing suspicion for CRN. After treatment with bevacizumab, interval resolution of vasogenic edema consistent with response of radiation necrosis was observed in coronal T2 FLAIR **(G)** and coronal T1 post contrast **(H)**. EBRT- external beam radiation therapy; SBRT- stereotactic beam radiation therapy; MRI- Magnetic resonance imaging; FLAIR- Fluid-attenuated inversion recovery; ORN- osteoradionecrosis; PENTOCLO protocol- One month of prednisone, amoxicillin/clavulanate, ciprofloxacin, and fluconazole followed by one month of pentoxifylline, alpha-tocopherol, prednisone, ciprofloxacin, and alendronate; CRN- Cerebral radiation necrosis.

One month after re-irradiation, he developed pain of the left hemicranium that was ultimately refractory to multiple and multimodal pain medications. The progressive and relentless nature of his pain prompted hospital admission three times. At that time, he described 10/10, constant pain of the left hemiface that was worsened with mastication and associated with photophobia, tinnitus, hemifacial hyperesthesia, and malodor. MR brain demonstrated nasopharyngeal mass with enhancement superiorly extending to the level of the foramen ovale and pre-styloid parapharyngeal space as well as the clivus and longus coli musculature (**Figures 1A, B**). Fiberoptic endoscopy was performed and demonstrated a lesion visually consistent with necrosis. The pathology of the biopsied lesion demonstrated squamous mucosa with underlying acute and chronic inflammation, granulation tissue, plasma cells, and atypical cells consistent with radiation atypia.

He was diagnosed with osteoradionecrosis of the clivus and treated with a modified PENTOCLO protocol (using alendronate instead of clodronate). He received one month of prednisone, amoxicillin/clavulanate, ciprofloxacin, and fluconazole before starting the modified PENTOCLO (pentoxifylline, alpha-tocopherol, and alendronate) with addition of prednisone and ciprofloxacin for one month. His pain dissipated precipitously and he demonstrated a sustained response at the time.

Two years later, surveillance imaging demonstrated progression, for which he was treated with additional carboplatin/5-fluorouracil followed by pembrolizumab. He remained stable for one year and then he presented with headaches and expressive aphasia; MR imaging showed a 1.6 x 1.3cm hemorrhagic mass at the left temporal lobe with associated vasogenic edema as well as residual nasopharyngeal carcinoma involving the left lateral nasopharynx, posterior nasal cavity, skull base, pterygopalatine fossa, posterior orbit, and cavernous sinus (**Figures 1C, D**). PET-CT demonstrated focal uptake in left nasal parapharyngeal region and clivus. Conclusion at the time was nasopharyngeal tumor recurrence with concerns for tumor extension to adjacent temporal lobe. However, there was reasonable doubt to suspect radiation injury and further review of brain MR imaging determined the temporal lobe lesions had no clear enhancing mass and borders were ill-defined, which was more consistent with cerebral radiation necrosis. He was treated with dexamethasone due to concerns for cerebral radiation necrosis and eight cycles of docetaxel for nasopharyngeal cancer recurrence with resolution of expressive aphasia.

The following year, surveillance imaging demonstrated invasion of the cavernous sinus and left trigeminal nerve concerning for disease progression, for which he was treated with carboplatin and gemcitabine. Soon after initiating this therapy, he developed a severe, unremitting headache, and imaging showed findings concerning for a left temporal lobe abscess. He was treated with empiric antibiotics and subsequent imaging demonstrated resolution. His headache improved but failed to resolve completely, and his treatment was switched to carboplatin and cetuximab for possible tumor progression.

**Figure 2:**
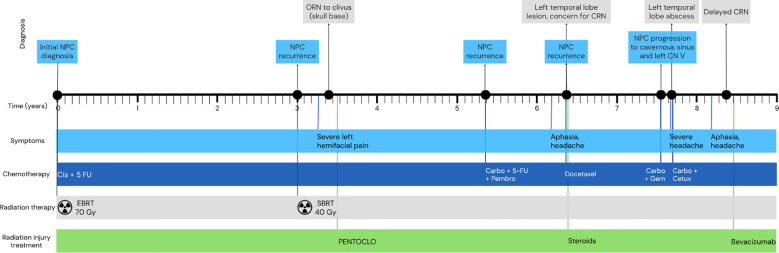
Timeline of disease progression since initial nasopharyngeal carcinoma diagnosis (representing year 0) through the years and different diagnosis. Carbo: Carboplatin; Cetux: Cetuximab; Cis: Cisplatin; CRN: Cerebral Radiation Necrosis; 5-FU: 5-fluorouracil; Gem: Gemcitabine; NPC: Nasopharyngeal Carcinoma; ORN: Osteoradionecrosis; Pembro: Pembrolizumab.

Six months later, he presented again with expressive aphasia and worsening headaches. His case and radiographs were reviewed at the hospital’s tumor board. MR brain demonstrated a lesion in the left temporal lobe with surrounding vasogenic edema (**Figures 1E, F**), and MRI perfusion did not show any hyperperfusion, which would have been found on active tumor. It was at this time that he was diagnosed with delayed CRN over 5 years after his last radiation session and treatment of osteoradionecrosis. He was treated with four total doses of bevacizumab 7.5mg/kg every three weeks, to which there was a marked response both clinically and radiographically (**Figures 1G, H**). A complete timeline of the patient’s progression can be seen in **Figure 2**.

## Discussion

This case presented a delayed diagnosis of CRN thought to be initially tumor progression. CRN is difficult to distinguish from tumor progression for several reasons. Presenting symptoms are primarily localization-related due to mass effect or loss of function and might include nausea, cognitive impairment, seizures, or as in our patient, headaches and expressive aphasia which can be seen in either tumor progression or CRN and a myriad of other neurological conditions (7, 8). Additionally, the timeframe within which CRN can occur is wide and often delayed anywhere between weeks to years after radiation therapy (5), such as in this patient who presented eight years after EBRT and five after SBRT.

Both ORN and CRN are part of the radiation-induced injury spectrum that can happen after radiation therapy for head and neck tumors. In both types, late injury involves the loss of parenchymal cells and vascular endothelial cells and a vicious cycle of tissue repair and fibrosis (9). However, the proposed pathophysiological mechanisms for each tissue are slightly different. Radiation-induced cerebral tissue injury is based on radiation injury to endothelial cells and apoptosis which releases oxygen free radicals and upregulation of hypoxia-inducible factor-1a (HIF-1α) and vascular endothelial growth factor (VEGF). This causes blood-brain-barrier (BBB) disruption, edema, fibrosis, vessel occlusion, and ischemia. Radiation injury to glial cells and immuno-inflammation with immune cell infiltration and secretion of inflammatory cytokines further disrupt BBB producing more hypoxia-induced necrosis and demyelination (10).

Osteoradionecrosis on the other hand has been explained through the radiation-induced fibrosis theory, which describes a fibroatrophic mechanism, in which reactive oxygen species and transforming growth factor-beta 1 (TGF-β1) induce inflammation with TNF-α and cytokine release, fibrosis, and tissue necrosis (11, 12). Osteocyte damage, absence of osteoblasts from bone margins, and lack of new osteoid is seen histologically (11, 12).

The uniqueness of this case is the history of osteoradionecrosis of the skull base followed by development of CRN several years later which represents a delayed response. There have been reports of osteoradionecrosis occurring concurrently with CRN (13, 14) but to the best of our knowledge this chronological delay in these two anatomical sites is a rare occurrence. Prior treatment for osteoradionecrosis of the clivus might have delayed CRN expansion and detection. The involvement of a different anatomic site rather than a local or contiguous area also might be due to latency of radiation-induced damage in all tissues previously exposed to it. Another possibility is inadequate treatment of osteoradionecrosis. At the time of osteoradionecrosis, our patient was treated with a modified version of the PENTOCLO protocol. A regular PENTOCLO protocol consists of a combination of pentoxifylline plus alpha-tocopherol with a boost of clodronate, which in our patient was switched to alendronate. This combination combines synergistic antioxidant and anti-fibrotic effects (anti-TNF-a activity, decreased cytokine cascade, reactive oxygen species scavenging, etc), and decreased bone resorption, tackling the main pathophysiological mechanisms involved in ORN (11). Patients are usually primed before treatment with a combination of oral disinfiltrating treatment with 20 mg prednisone plus 2 g amoxicillin-clavulanate plus 1 g ciprofloxacin plus 50 mg fluconazole to allow further PENTOCLO penetration, which our patient also received (15).

Switching from clodronate to alendronate could theoretically have affected the effectiveness of the PENTOCLO protocol as clodronate has been described to have anti-inflammatory properties whereas alendronate has pro-inflammatory properties (16), but these effects would have been noticed earlier, and our patient did have improvement of his symptoms at the time. Long-term outcome data for the PENTOCLO protocol is limited but demonstrates precipitous recovery in more than 60% of patients and complete resolution without need for further intervention in about 30% (17). In our patient, the duration of treatment for osteoradionecrosis was less than has typically been evaluated. In a phase II trial, the duration of therapy was at least 6 months, and in a meta-analysis, the average duration was upwards of one year of therapy (15, 17, 18). However, this is less likely to have accounted for his later development of CRN as the radiation changes developed at an anatomic site distinct from the site of osteoradionecrosis rather than contiguously, meaning that if this short-term course of treatment had had any implication it would have caused recurrent osteoradionecrosis, not CRN. Moreover, though our patient was treated for one month, he did demonstrate resolution of his symptoms at the time.

Steroids, specifically Dexamethasone, which provides great brain tissue penetration, are first-line treatment for CRN but off-label use of Bevacizumab, a VEGF-inhibitor, has demonstrated improved symptomatic relief and radiographic response by targeting endothelial cells thus decreasing blood brain barrier permeability and edema with less side effects compared to steroids (19). The use of Bevacizumab at doses ranging between 5–10 mg/kg every 2–3 weeks for 2–4 cycles has a good level of evidence for treating symptomatic CRN (20). In our case, the patient had a resolution of symptoms and imaging findings with bevacizumab, further aiding the diagnosis of CRN.

Various imaging modalities can help distinguish CRN from tumor progression. Magnetic resonance (MR) is readily available in most centers, and CRN typically demonstrates a ring-enhancing lesion on T1-sequences and hyperintense signals on T2-fluid attenuated inversion recovery (FLAIR) sequences, but these can also be seen in tumor progression (1). White matter lesions, contrast-enhanced lesions, and cysts are also common findings for delayed CRN (21). Apparent diffusion coefficient (ADC) values in neoplastic processes are reduced compared to necrosis due to rapid cell proliferation in the former and decreased cell density in the latter (1). Perfusion-weighted imaging demonstrates increased relative cerebral blood volume (rCBV) coefficients in tumorigenesis (≥2.5) and decreased values in CRN (<0.6) (1, 2), which was consistent with the imaging findings in our patient. Positron emission tomography (PET) scans additionally have potential in distinguishing the two but can be equivocal in the setting of low-grade neoplasms or when there is concurrent tumor progression and necrosis, which was also the case of our patient at the time first suspicion for CRN was raised (1, 2). A systematic review and meta-analysis concluded that MR spectroscopy and single photon emission computed tomography (SPECT) carry greater utility than MRI to distinguish CRN from tumor progression (22). More recently, an umbrella review and network meta-analysis demonstrated that nuclear imaging techniques such as PET FET and DOPA PET exhibit better reliability when discriminating between the two entities, but these might not be always available (23).

CRN was most likely present at the patient’s initial presentation with headaches and expressive aphasia (**Figures 1C, D**). At the time, he was treated with both chemotherapy for possible tumor recurrence and steroids as there was reasonable suspicion for CRN, which could explain why his subsequent imaging demonstrated temporary resolution. It was only after his headache and expressive aphasia recurred while on chemotherapy but not on steroids, with redemonstration of similar imaging findings that he was finally diagnosed with delayed CRN and treated with a steroid sparing-agent (**Figures 1E, F**).

This patient developed CRN without interval change in residual nasopharyngeal carcinoma or treatment-related changes already present in the nasopharynx and skull base. It is our belief that both radiation-induced injuries were triggered at the same time with faster development of symptoms to immediate target areas as in our patient in whom symptoms of osteoradionecrosis developed first as it is our assumption that area received higher amounts of radiation being closer to the initial tumor.

## Conclusion

This case illustrates the diagnostic challenge of CRN and the importance of keeping CRN on the differential for patients with a history of radiation therapy to the head or neck with new or persistent neurological symptoms even years after last radiation therapy. Radiation necrosis may progress to affect other anatomical sites even if prior sites were successfully treated and radiographic improvement was noted. Moreover, the role of re-irradiation in disease progression and extension should be considered. A field limitation neuro-oncologists usually face is the challenge of reaching a pathological diagnosis without access to brain tissue, which as in this case may delay therapy. Clinical acuity and comprehensive imaging review using different available modalities should help guide the diagnosis. Moreover, understanding the different pathophysiological mechanisms involved in radiation injury to different organs can orient management.

## Limitations

As we herein report a singular case, we cannot determine a cause-and-effect relationship and the extent to which our findings are generalizable. Our patient reached our clinic many years after his initial diagnosis of nasopharyngeal carcinoma and radiation treatment. Radiation therapy records were never disclosed to us. Thus, our conclusions are inferential in regards of target volumes involved during initial radiation and recurrence, the dose constraints at time of radiation planning, or cumulative doses to different tissues involving skull base and temporal lobes.

## Data Availability

The original contributions presented in the study are included in the article/supplementary material. Further inquiries can be directed to the corresponding author.
